# Siloxane Molecules: Nonlinear Elastic Behavior and Fracture Characteristics

**DOI:** 10.1021/acs.macromol.2c02576

**Published:** 2023-02-08

**Authors:** Tianchi Li, Eric R. Dufresne, Martin Kröger, Stefanie Heyden

**Affiliations:** † Soft and Living Materials, Department of Materials, ETH Zurich, CH-8093 Zurich, Switzerland; ‡ Polymer Physics, Department of Materials, ETH Zurich, CH-8093 Zurich, Switzerland; § Magnetism and Interface Physics, Department of Materials, ETH Zurich, CH-8093 Zurich, Switzerland

## Abstract

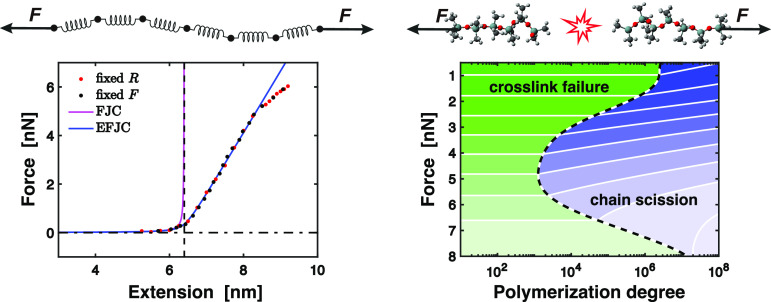

Fracture phenomena in soft materials span multiple length and time scales. This poses a major challenge in computational modeling and predictive materials design. To pass quantitatively from molecular to continuum scales, a precise representation of the material response at the molecular level is vital. Here, we derive the nonlinear elastic response and fracture characteristics of individual siloxane molecules using molecular dynamics (MD) studies. For short chains, we find deviations from classical scalings for both the effective stiffness and mean chain rupture times. A simple model of a nonuniform chain of Kuhn segments captures the observed effect and agrees well with MD data. We find that the dominating fracture mechanism depends on the applied force scale in a nonmonotonic fashion. This analysis suggests that common polydimethylsiloxane (PDMS) networks fail at cross-linking points. Our results can be readily lumped into coarse-grained models. Although focusing on PDMS as a model system, our study presents a general procedure to pass beyond the window of accessible rupture times in MD studies employing mean first passage time theory, which can be exploited for arbitrary molecular systems.

## 1 Introduction

Most things in life start small. This basic concept also applies to the failure of soft materials, emerging from the rupture of interatomic bonds. Predicting the fracture journey that follows becomes a question of failure mechanisms and length scales.^1−3^ In view of failure mechanisms, Lake–Thomas theory has formed our understanding of how much energy it takes to break an elastic chain.^4^ When a crack propagates within a stretched elastic material, each repeat unit within chains crossing the fracture plane stores energy. The resultant fracture energy should thus reflect the elastic energy stored within the entire chain instead of pure single bond scission. Recent works on tough hydrogels hint at a more complicated picture, in which network characteristics such as entanglements have a crucial effect on fracture.^5,6^

Multiple length scales form the basis of the classical fracture mechanics picture. In the ideally brittle limit, dissipation and material failure occur on the scale of the atomistic separation length. In soft tough materials, the characteristic length scale in the continuum limit is the so-called *elasto-adhesive* length. This length scale is typically microscopic and represents the region of nonlinear elastic deformation around a macroscopic crack tip.^3^ It can be coupled to molecular failure processes at small scale^4,7^ as well as energy dissipation at the mesoscale^8−10^ and macroscopic effects such as crack blunting.^11,12^ In a recent work, scale-free cavity growth at constant driving pressure was accessed in the mesoscopic region.^13^ In this picture, no well-defined crack tip exists and corresponding process zones for the calculation of fracture energies becomes obsolete.

Fracture in soft solids thus displays manifold characteristics that are deviating from classical theories. To get further insight into what governs these deviations, multiple length and time scales need to be bridged, which poses a major computational challenge. Here, we address this challenge by providing a detailed description of the nonlinear elastic response of molecular building blocks up to fracture. These building blocks can then provide starting grounds for higher level coarse grained models.

Previous studies on the force–extension relation and fracture of individual molecules encompass both experimental and computational investigations. Experimental studies include atomic force microscopy (AFM),^14−17^ optical tweezers,^18−20^ and magnetic tweezers.^21−23^ Due to its large accessible force range up to nN*)* and high resolution, AFM has been widely adopted.^24^ Investigations using AFM comprise a wide spectrum, ranging from proteins,^25,26^ DNA,^27,28^ polysaccharides,^29^ poly(ethylene glycol),^30^ and poly(methacrylic acid)^31^ to polydimethylsiloxane (PDMS).^32^

Using computational methods, ab initio molecular dynamics (AIMD)^33,34^ simulations allow for an on-the-fly computation of electronic structures based on quantum mechanics. While bond fracture can be modeled in this setting, high computational costs limit AIMD studies to nm*)* and ps*)*.^35−37^ At higher length and time scales, steered molecular dynamics (MD) simulations have emerged as the primary method in studying the force–extension behavior of molecules.^38−41^ Classical MD methods are amenable of treating system sizes of several hundreds of nanometers and time scales on the order of nanoseconds. However, atomic interactions are typically modeled via empirical interatomic potentials, which require a predefined atomic connectivity remaining unchanged throughout simulations, such that fracture of interatomic bonds cannot be described. As an alternative, bond-order-based force fields were developed to bridge the gap between ab initio and empirical force fields. Here, we derive the quasi-static force–extension and rupture properties of single molecules up to  and  ns) by enriching all-atom steered molecular dynamics simulations with a bond-order-based force field (ReaxFF),^42−46^ with the help of the LAMMPS software package.^47^ Unlike classical atomistic bond potentials, ReaxFF allows for different atomic bonding states, such that fracture events can be captured. Simulations thus reduce the gap between length and time scales accessible using ab initio computational methods and experimental approaches. We focus on PDMS as a model system as used in previous studies,^13^ for which both linear PDMS and cross-linked PDMS are investigated.

## 2 Molecular Dynamics Studies

### 2.1 Nonlinear Elastic Response

**Figure 1:**
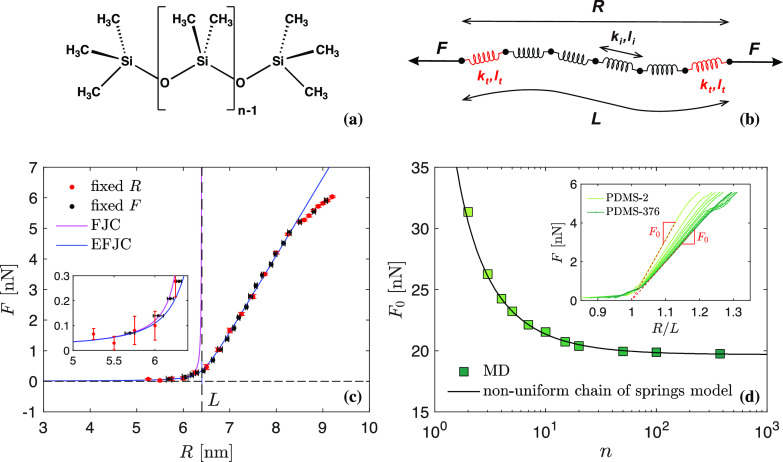
(a) Chemical structure of PDMS-*n* illustrating (*n* – 1) repeating units. (b) Nonuniform chain of springs model consisting of two types of Kuhn springs. (c) Force–extension relation *F*(*R*) of a single PDMS-27 molecule measured at *T* = 300 K and *P* = 1 atm in the presence of HMDSO solvent molecules. Red/black data points are obtained in a displacement/force controlled setting, respectively. Inset showing the small extension regime (*R* < *L*). The FJC model (pink solid, upper line) and elastic FJC model (blue solid, lower line) both capture the entropic regime *R* ≪ *L*, while the latter shows better agreement in the enthalpic region *R* > *L*. (d) Evolution of *F*_0_ = *L* ∂*F*/∂*R*, obtained at *T* = 1 K within the elastic regime as shown in the inset for *n* = 2 up to *n* = 376. MD data are captured well by a model of nonuniform chain of springs, consisting of two types of Kuhn springs as sketched in (b).

Prior to failure, the static molecular response is governed by entropic elasticity at extensions well below the unstretched contour length and enthalpic elasticity at higher extensions. The exact shape of this nonlinear elastic force–extension relation depends on the specific molecular structure under investigation. To derive the nonlinear elastic response of siloxane molecules, PDMS-*n* molecules of varying polymerization degree *n* are created. Figure 1a illustrates the chemical structure of PDMS-*n* as an example. Each PDMS-*n* molecule is embedded in a simulation box, which is set up both with and without solvent molecules. When solvent molecules are present, periodic boundary conditions are applied. We use hexamethyldisiloxane (HMDSO) molecules as a solvent, as interactions between HMDSO and PDMS do not alter the rupture behavior of PDMS (compared to interactions with itself).^37^ In comparison, trace amounts of water were found to lower the maximally attained rupture stretch.^37^

All simulations are performed with the parameter set specifically trained and optimized for PDMS.^43^ Without solvent molecules, the number of degrees of freedom is 3*n*_a_, with *n*_a_ being the number of atoms in PDMS-*n. n*_a_ scales linearly with polymerization degree *n*. For simulations in which solvent molecules are present, the overall system size increases by 3*n*_s_ degrees of freedom based on *n*_s_ solvent molecules. There is no upper constraint on *n*_s_. Its lower bound is set by the requirement of generating sufficiently large RVE′s for subsequent steered molecular dynamics runs, preventing self-interactions. For simulations of PDMS-27 up to fracture, *n*_s_/*n*_a_ ∼ 60 amounts to 60 × 3*n*_a_ degrees of freedom. Here, the multiplicative factor of 60 stems from the presence of solvent molecules. In all simulations, a time step of 0.1 fs is applied. Systems are relaxed in an NPT ensemble at ambient conditions (*T* = 300 K, *p* = 1 atm). Following relaxation, a constant repulsive force *F* between the two terminal Si atoms is applied in an NVT ensemble. In this ensemble, volume *V* is held constant, such that the equilibrated end-to-end distance is purely based on the applied force (rescalings of the simulation box are prohibited). Results are compared to a displacement controlled setting, in which both terminal Si atoms are held constant at fixed end-to-end distance *R* and the exerted force is recorded. Figure 1c shows the nonlinear elastic reponse of PDMS-27 up to fracture. The choice of boundary condition does not influence the force–extension relation in both entropic (*R* ≪ *L*) and enthalpic (*R* > *L*) regimes, where the unstretched contour length *L* marks the crossover point. For comparison with classical polymer models, the inset of Figure 1c highlights the divergence of the freely jointed chain model (FJC)^21^ for an end-to-end chain distance *R* approaching the unstretched contour length *L* = 6.4 nm. In contrast, the elastic freely jointed chain model (EFJC)^48^ captures the nonlinear elastic force–extension relation also within the enthalpic regime *R* > *L*. The change of slope at large forces is encoded in the *F*-dependent bond potential of mean force *U*(*b*;*F*), as investigated in more detail in Section 2.2.

The force–extension curve at large deformation is linear. As expected from the EFJC model, the force–extension relation of a single polymer chain is given as
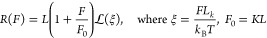
1Equation 1 represents a classical FJC model with an added elastic extension (1 + *F*/*F*_0_). Within the entropic regime, elasticity is modeled via the Langevin function . With the added elastic extension, the EFJC model introduces the effective Hookean spring constant *K* as an additional elastic parameter within the enthalpic regime.

For computational efficiency, solvent molecules are removed for the determination of *F*_0_, and *T* = 1 K is chosen to reduce thermal noise. All other simulations are performed at *T* = 300 K. Note that in this study we focus on the enthalpic regime, in which temperature effects on the mechanical response become negligible. Insensitivity of *F*_0_ toward both temperature and solvent molecules in the enthalpic regime is tested for short oligomers (see Figures S3 and S4 in the Supporting Information). We find that both temperature and solvent molecules do not influence *F*_0_ in the enthalpic regime. For small forces *F* ≪ *F*_0_ in the entropic limit, we have *R*(*F*) ≃ *F*/*K̃*, with *K̃* = 3*k*_B_*T*/(*LL*_*k*_) the elastic Hookean spring constant within the entropic regime. For large forces *F* ≫ *F*_0_ in the enthalpic limit, *R*(*F*) ≃ *F*/*K*, as the Langevin function  approaches unity for ξ ≫ 1. *L*_*k*_ is obtained from fitting the EFJC model to the measured force–extension curve at *T* = 300 K (see Figure 1c). A comparison to other hypothetical Kuhn segments (which consistently overpredict the unstretched contour length *L*) is shown in Figure S2). Further support giving an independent estimate of *L*_*k*_ from analyzing the Si–Si vector correlation function is given in Supporting Information Section S2. Here, *L*_*k*_ = 5.5 ± 0.7 Å is the Kuhn length of the polymer chain corresponding to the mean end-to-end distance of a Si–O–Si–O–Si triplet, which in the following is abbreviated as Si–Si–Si.

For a low polymerization degree of *n* = 2, the resultant slope *F*_0_ when plotting force *F* versus stretch *R*/*L* attains its maximum value *F*_0_ = 31.35 nN (see the inset in Figure 1d). *F*_0_ decreases with increasing *n* in a nonlinear fashion as illustrated in Figure 1d. Characteristic forces are calculated for a large range of polymerization degrees *n* ∈ [2, 3, 4, 5, 7, 10, 15, 20, 50, 100, 376]. This differs from a classical model of *n* identical springs in series, for which *K* ∝ 1/*n* and *L* ∝ *n*. To determine the source of this deviation, we track bond length distributions at fixed repulsive force between terminal Si atoms. We find that within the enthalpic regime internal triplet distances are shorter than terminal ones. This difference in triplet distance distributions can be related to restrictions in bond angles and dihedrals at terminal atoms. Endowing internal Si–Si–Si Kuhn segments with spring stiffness *k*_*i*_ and equilibrium length *l*_*i*_, whereas terminal Kuhn segments possess spring stiffness *k*_*t*_ and equilibrium length *l*_*t*_, gives the overall Hookean spring constant *K* and contour length *L* as
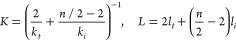
2Here, *n*/2 denotes the total number of Kuhn segments, where *n* is the polymerization degree. *k*_*t*_ = 47.3 ± 0.1 nN/nm and *l*_*t*_ = 0.52 ± 0.01 nm are directly computed from the bond length distribution of PDMS-4, which only consists of two terminal Kuhn segments (*k*_*t*_ = 2*K* and *l*_*t*_ = *L*/2). Using a fit to simulation results for PDMS-5 to calculate the remaining free parameters, we find that *k*_*i*_ = 40.82 ± 0.01 nN/nm and *l*_*i*_ = 0.48 ± 0.01 nm of the internal Kuhn segment. As shown by the solid black line in Figure 1d, this model of a nonuniform chain of springs agrees well with MD data and captures the effect of terminal springs at small *n* as well as convergence of *F*_0_ to a plateau at large *n*.

To summarize, the elastic response of PDMS oligomers (within both entropic and enthalpic regimes) is characterized by the Si triplet length *L*_*k*_ based on two reasons: First, the entropic part of the force–extension curve suggests *L*_*k*_ to be identical with the extension of a Si–Si–Si triplet. Second, *n* dependencies of *K* and *L* are consistently captured only if the number (*n*/2) of Si–Si–Si triplets is used in eq 2. In sharp contrast, the fracture behavior to be discussed next will be dominated by the *F*-dependent characteristics of single covalent atomic bonds.

### 2.2 Fracture Characteristics

At the molecular scale, fracture is stochastic. An intuitive question to ask is, “where and when does a network tend to break?”. Here, we try to quantitatively answer this question for PDMS in terms of mean rupture times and preferred fracture modes.

To distinguish between rupture of the PDMS-*n* backbone (*chain scission*) and rupture at cross-linking sites (*cross-link failure*), we take into account two different structures: PDMS-*n* as used in the previous section as well as two PDMS-4 molecules linked via a cross-linking site −CH_2_–CH_2_. Chain scission thus stems from the rupture of Si–O bonds, while cross-link failure results from rupturing Si–C bonds. The accessible window of mean rupture times in MD studies lies in the range of 10^–2^–10^0^ ns. The upper limit is set by computational feasibility, while the lower limit depends on the molecular vibration frequency of the polymer chain below which inertial effects dominate the response.

**Figure 2:**
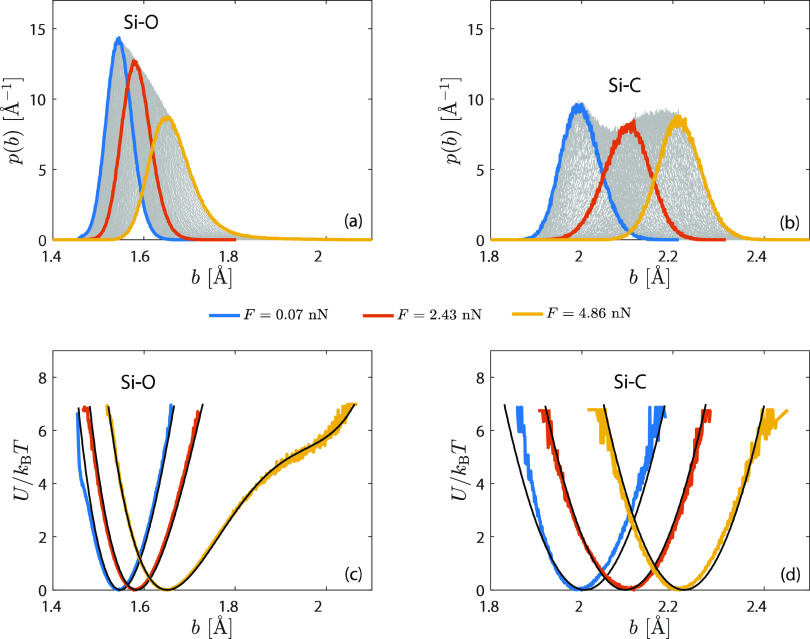
(a, b) Stationary nonequilibrium MD probability densities of Si–O and Si–C bond lengths on linked PDMS-4 at various force levels (simulations performed in the presence of solvent molecules). Gray curves denote intermediate force levels. (c, d) Radial bond potential of mean force calculated from bond length distributions highlighted in (a, b). Black curves represent polynomial fits of order 4 (Si–O) and 2 (Si–C). All simulations are performed at *T* = 300 K.

To extend beyond this rupture time window and determine mean bond rupture times τ(*F*) on longer time scales, we use a statistical extrapolation scheme. Our approach renders a close analogy to the calculation of mean first passage times for chemical processes with a single reaction coordinate,^49^ for the thermal or enforced breakage of discrete one-dimensional chains,^50^ Morse chains,^51^ and biomolecules.^52^ Its derivation is provided in the Supporting Information. We proceed in the following way: Stationary equilibrium Si–O and Si–C bond length probability densities *p*(*b*) of PDMS-4, PDMS-376, and linked PDMS-4 are measured at different levels of constant force (cf. Figure 2). Using *p*(*b*), we calculate mean chain rupture times, for which we need to pass from rupture times of single bonds to those of chains with 2*n* bonds.

At fixed force, directly measured single bond probability densities *p*(*b*) serve to define an effective potential, with
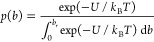
3Here, *b*_*r*_ is the rupture bond length. For later calculations of mean chain rupture times, we need a functional form of the effective bond potential *U*. In the following, using the notation *U*(*b*;*F*), we emphasize that the potential is a function of bond length *b*, while its parameters (in this case *b*_1_) depend on *F*. Numerically solving for *U* from eq 3 at different levels of applied force, we see that *U* needs to satisfy the following properties: It should have the generic form of a double-well potential (fourth-order polynomial) with minima corresponding to two different equilibrium bond lengths (cf. Figure S7). Here, we denote *b*_1_ as the equilibrium bond length corresponding to the first minimum and choose *U*(*b*_1_) = 0 for convenience. Most importantly, we observe a nearly *F*-independent parabolic shape of *U*″(*b*) about its second minimum at *b* ≃ *b*_2_, i.e., *U*″(*b*) = *c*_2_ + *k*_2_(*b* – *b*_2_)^2^ (cf. Figure S7). Because *U*″(*b*) exhibits a parabolic and *F*-independent shape and location, the corresponding parameters *b*_2_, *c*_2_,and *k*_2_ can be treated as *F*-independent constants. This finding forms the basis of rendering our statistical extrapolation scheme feasible because *b*_1_(*F*) remains as the only force-dependent fitting parameter. In addition, we only observe a weak dependence of *b*_1_ on *F*, which forms the basis for an extrapolation to higher force regimes. These observations lead to a functional form of *U*(*b*;*F*) as
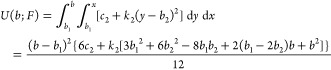
4Bond length distributions (Figures 2a,b) are fitted to eqs 3 and 4 with fitting parameters given in Table S1. The resulting radial bond potentials of mean force are illustrated in Figure 2c,d, with corresponding polynomials of order 4 (Si–O bond) and order 2 (Si–C bond, for which *b*_2_ = *k*_2_ = 0). Note that fitting deviations in Si–C potentials are based on restricting *b*_1_(*F*) to be the only force-dependent parameter. The largest possible instantaneous Si–O an Si–C bond length value in stable chain configurations is given in Table S1. As shown in Figure 2c, an increasing nonlinearity develops with increasing tension for Si–O bonds. This is rooted in bond angle potentials losing their dominance within the energy landscape due to the externally enforced alignment. At this point, the remaining interactions (dihedral, Si–C, Si–H, O–H) come into play.

With an expression for *U*(*b*;*F*) at hand, we proceed with the calculation of mean chain rupture times, passing from rupture times of single bonds to those of chains with 2*n* bonds. Furthermore, it needs to be verified that a theory neglecting inertia effects captures the attendant fracture characteristics.

Neglecting inertia effects, the mean rupture time of a single bond is calculated from the Fokker–Planck equation as^53−55^
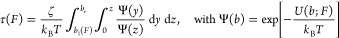
5This is a purely theoretical limit, as atomistic simulations (PDMS chains consist of multiple Si–O and Si–C bonds) measure τ_*n*_. ζ is an a priori unknown friction coefficient, which will be determined later by matching theoretical rupture times τ_*n*_(*F*) with those obtained from MD simulations. Utilizing the Fokker–Planck approach, eq S-2 (or equivalently Brownian dynamics simulations via eq S-1) can be used to explore the rupture time distribution *p*(*t*_*r*_;*F*) of a single bond, which is nearly monoexponential (apart from a small dip at *t*_*r*_ → 0).

**Figure 3:**
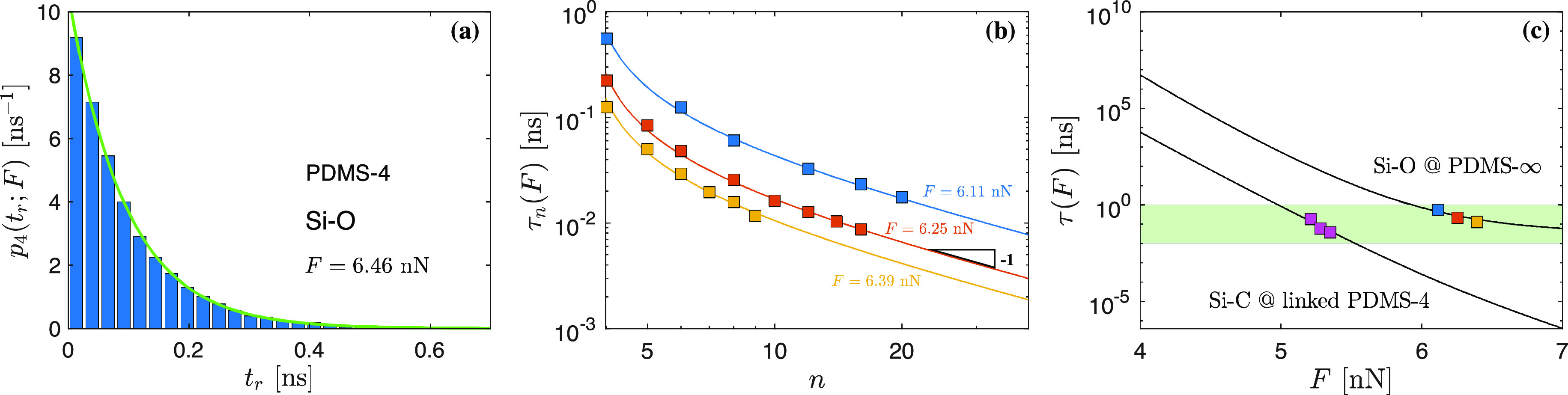
(a) Measured rupture time probability density *p*_4_(*t*_*r*_;*F*) of PDMS-4 at constant stretching force *F* = 6.46 nN. Green solid line: monoexponential used in the calculation of mean rupture time τ_*n*_(*F*). (b) Evolution of mean chain rupture time τ_*n*_(*F*) with increasing polymerization degree *n* at three different forces. Each data point is the average of 10000 simulations. Solid lines are obtained from fitting the functional form given in eq 7. For large *n*, τ_*n*_(*F*) ∝ 1/*n* as expected. Deviations for short chains are related to the effect of nonuniform chain of springs highlighted in Figure 1d. (c) Single-bond mean rupture time τ(*F*) for Si–O (obtained from eq 7) and Si–C (average of individual MD simulations). Solid lines: solution to the mean first passage time problem (5) based on *U* (cf. Figure 2b,c). The shaded region highlights the rupture time window accessible in MD studies. All simulations are performed at *T* = 300 K.

In order to pass to the rupture time distribution *p*_*n*_(*t*_*r*_;*F*) of a chain with polymerization degree *n* (which thus contains 2*n* bonds), we assume independent bonds. The probability of a chain (i.e., at least one of its assumed identical bonds) rupturing during time interval *t* after onset of *F* at time *t* = 0 is
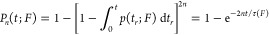
6The term in parentheses in eq 6 denotes the probability of an individual bond staying intact until time *t*. The probability distribution for rupture times *t*_*r*_ of *n*-chains (PDMS-*n*) is thus *p*_*n*_(*t*_*r*_;*F*) = (d/d*t*_*r*_)*P*_*n*_(*t*_*r*_;*F*) = 2*n*e^–2*nt*_*r*_/τ(*F*)^/τ(*F*), from which the mean chain rupture time τ_*n*_(*F*) follows as τ_*n*_(*F*) = τ(*F*)/2*n*. We compare these theoretical expressions to those measured in MD simulations. Figure 3a shows measurements on PDMS-4. MD measurements show a monoexponential shape of *p*_4_(*t*_*r*_;*F*), which is in agreement with the Fokker–Planck prediction for a single bond and the assumption of independent bonds in chains of higher polymerization degree.

Figure 3b illustrates τ_*n*_(*F*) at three different constant stretching forces *F*, for which each data point is the average of 10000 independent samples. For large *n*, τ_*n*_(*F*) approaches the expected ∝1/*n* limit. Deviations from this scaling for short chains are reminiscent of the nonuniform chain of springs effect highlighted in Figure 1b. Equivalent to the functional form given in eq 2, we have
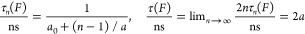
7Fitting parameters at *F* = 6.25 nN are obtained as *a*_0_ = −24 ± 1 and *a* = 0.108 ± 0.004, while at *F* = 6.11 nN, *a*_0_ = −9.1 ± 0.4, and *a* = 0.28 ± 0.01. Single bond mean rupture times depicted in Figure 3c are calculated as τ^Si–O^(*F*) = lim_*n* → ∞_2*n*τ_*n*_(*F*) = 2*a* ns. For Si–C bonds (which are present twice in linked PDMS-4), force levels of *F* = 5.21 nN, *F* = 5.28 nN, and *F* = 5.35 nN are investigated. This force range in MD already spans 2 decades in single-bond mean rupture time τ(*F*). The resulting single bond mean rupture time is computed as τ^Si–C^(*F*) = 2τ^linked-PDMS-4^(*F*).

By matching the measured τ_*n*_ with the theoretically predicted one, we can furthermore determine ζ (which is the shape-preserving, force-independent vertical shift required to match measurement and theory). Solid lines in Figure 3c illustrate the τ(*F*) resulting from the Fokker–Planck equation (5). This solution allows to extend beyond the rupture time window accessible in MD studies (shaded region in Figure 3c). We find that the lifetime of a single representative Si–O bond is much longer than that of a Si–C bond, with an increasing gap for larger *F* (based on the significant difference in potentials at high forces).

**Figure 4:**
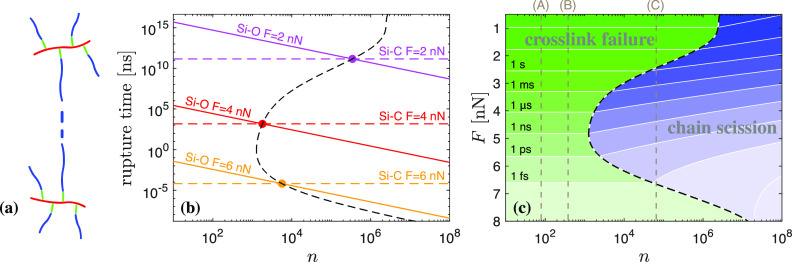
Cross-link failure versus chain scission. (a) We model constituent molecules of monodisperse strands, testing for chain scission along the PDMS backbone (blue) versus failure at cross-linking junctions (green). (b) Evolution of crossover polymerization degree *n*_*c*_ (dashed black line) with increasing force *F*. Intersections of τ_*n*_(*F*) for Si–O along the backbone and τ_2_(*F*) for Si–C at two cross-linking junctions are illustrated at three different force levels. (c) Effective rupture time of a network strand (contour lines) as a function of *n* and *F*. Green: region dominated by failure of cross-linking junctions. Blue: chain scission dominated region. (A) Sylgard 184 (*n* = 78), (B) DMS-V31 (*n* = 376), and (C) Sylgard 186 (*n* = 64848).^60^ All predictions apply to *T* = 300 K.

To determine preferred fracture mechanisms, we note that with increasing polymerization degree *n*, τ_*n*_^Si–O^(*F*) decreases, while τ_*n*_^Si–C^(*F*) is constant (based on the constant number of Si–C bonds when focusing on the single chain level; see Figure 4b). Rupture of Si–O bonds (chain scission) and Si–C bonds (cross-link failure) thus becomes comparable at a crossover polymerization degree *n*_*c*_. Figure 4b displays a comparison of mean chain rupture times τ_*n*_(*F*) at different levels of applied force. Tracking the crossover polymerization degree *n*_*c*_ allows to identify two different failure regimes: For *n* < *n*_*c*_, cross-link failure is anticipated, while chain scission is the preferred failure mode for *n* > *n*_*c*_. Figure 4c highlights the effective rupture time (taking into account both Si–O and Si–C bonds) as contour lines as a function of *n* and *F*. Again, the crossover polymerization degree *n*_*c*_ differentiates a region dominated by cross-link failure (green) from a regime dominated by chain scission (blue).

We observe a strengthening effect in Si–O bonds with increasing force, which is reminiscent of phenomena observed in systems involving catch bonds, e.g., membrane-to-surface adhesion,^56^ myosin and actin,^57^ or signaling receptors and their ligands.^58,59^ This strengthening emerges as a “re-entrant” effect of cross-link failure for polymerization degrees *n* > 10^3^, which can be related to the higher order structure of Si–O bond potentials (see the nonlinearity developing at higher forces, Figures 2, S5, and S9). Si–O bonds are stable at low forces, at which the failure of cross-linking junctions is the dominating fracture mechanism. At intermediate forces, chain scission dominates. At high forces, at which the increasing stiffness of the second minimum in Si–O potentials comes into play, fracture characteristics are dominated by cross-link failure again. With increasing *n*, the force regime dominated by chain scission grows, which is in keeping with eq 6.

Typical siloxane materials used in the laboratory setting are highlighted in Figure 4c in terms of polymerization degree *n*. Single molecules in Sylgard 184 and DMS-V31 are entirely dominated by cross-link failure. In contrast, individual molecules in Sylgard 186 feature a much higher polymerization degree. As such, their fracture behavior strongly depends on the applied force, with cross-link failure in the low force regime transitioning to chain scission at *F* > 2.5 nN.

In the above, we model rupture time distributions using an inertia-free Fokker–Planck approach. To justify this approach, it remains to investigate the absence of solvent molecules on rupture time distributions *p*_*n*_(*t*_*r*_,*F*). While the dynamics of bond lengths exhibits inertia effects, we find the rupture time distribution to be unaffected by the presence/absence of explicit solvent molecules, as shown in Figure S8. Without solvent molecules, inertia effects are maximal and friction is absent. The presence of solvent molecules provides additional noise and stochastic collisions, such that inertia effects are diminished. The rupture time distribution is thus also unaffected by the degree of suppression of inertia effects. Furthermore, we observe a nearly monoexponential rupture time probability distribution, in which the majority of bonds does not break during the first oscillation but at later times. This is in stark contrast to the dominance of inertial effects, for which bonds that are still intact after the first oscillation would never fail. (The rupture criterion would not be fulfilled in subsequent oscillations if it was not already fulfilled in the first oscillation.)

## 3 Discussion and Outlook

This work characterizes the nonlinear elastic and fracture behavior of PDMS. The nonlinear elastic response of siloxane oligomers is captured well using the EFJC model, within both entropic and enthalpic regimes. At low polymerization degrees *n*, we find a deviation from a classical scaling of effective stiffness *K* ∝ 1/*n*, which can be captured with a simple model of nonuniform chains of springs, with each spring constituting a Kuhn segment.

Passing to the inelastic behavior of PDMS, we focus on rupture times of both Si–O bonds (present in the backbone of siloxane oligomers) as well as Si–C bonds (present at cross-linking sites). When calculating mean chain rupture times of siloxane oligomers, we again observe a deviation from classical scalings (τ_*n*_(*F*) ∝ 1/*n*) at low polymerization degrees. Similar to the nonlinear elastic part, a model including the “end effect” on mean chain rupture times agrees well with simulations.

We find that the lifetime of single Si–O bonds is much longer than that of a Si–C bond. We define a crossover polymerization degree *n*_*c*_ at which the dominating rupture mechanism of the cross-linked network passes from cross-link failure (rupture of Si–C bonds) to chain scission (rupture of Si–O bonds on PDMS-*n*). To pass to long time scales, we use a statistical extrapolation scheme in order to understand bond fracture within the network. Surprisingly, we find that the dominating fracture mechanism depends on the applied force scale in a nonmonotonous fashion. The nonmonotonic dependence of *n*_*c*_ on *F* is rooted in nonlinearities in the Si–O bond potential and the corresponding stiffness dependence on force at the two minima.

For single molecules in typical siloxane materials used in the laboratory setting, such as Sylgard 184 and DMS-V31, our analysis suggests breakage exclusively at cross-links. For individual molecules in Sylgard 186 featuring higher polymerization degrees, however, the attendant failure mode depends on the magnitude of applied force. While cross-link failure dominates at low forces, chain scission is expected for *F* > 2.5 nN. It furthermore bears mentioning that our analysis focuses on the single chain level. Possible inhomogeneities in force distribution based on network topology (and the resultant changes in fracture characteristics) furnish an important point for future studies. These could aid in elucidating network properties passing from the single chain to the continuum level, focusing on the influence of polymerization degree and cross-link density.

Our results provide building blocks, which can be readily used in coarse-grained higher scale models. As an example, network models of nonlinear springs could be easily tailored to siloxane systems by implementing a material model corresponding to the nonlinear elastic response derived in this work. In this setting, cross-linking molecules could be lumped into nodes representing cross-linking sites. Tracking attendant forces upon deformation would then allow for the implementation of fracture criteria corresponding to those derived in this work.

Finally, our work provides a generic procedure to pass beyond the window of accessible rupture times in MD studies, which can be applied for arbitrary molecular systems.

## Supporting Information Available

(i) Characteristic force at finite temperature, (ii) Kuhn length, (iii) parameters of the double well potential, (iv) mean bond rupture time, (v) influence of boundary conditions, (vi) bond length distributions and potentials, (vii) rupture times with and without HMDSO solvent, and (viii) mean chain rupture times for different polymerization degrees (PDF)
