# Supplementary material for: Siloxane Molecules: Nonlinear Elastic Behavior and Fracture Characteristics
Source: Macromolecules. 2023 Feb 8;56(4):1303–10. doi: 10.1021/acs.macromol.2c02576 (PMC9979691; doi:10.1021/acs.macromol.2c02576)
Supplement: Supplementary file 1 — ma2c02576_si_001.pdf [file ma2c02576_si_001.pdf]

SUPPLEMENTARY INFORMATION

Siloxane molecules: Nonlinear elastic behavior and fracture characteristics

Tianchi Li[1], Eric R. Dufresne[1], Martin Kröger[2,3], Stefanie Heyden[1*)]

[1)] Soft and Living Materials, Department of Materials, ETH Zurich, CH–8093 Zurich, Switzerland

[2)] Polymer Physics, Department of Materials, ETH Zurich, CH–8093 Zurich, Switzerland

[3)] Magnetism and Interface Physics, Department of Materials, ETH Zurich, CH–8093 Zurich, Switzerland

[*)] Corresponding author: stefanie.heyden@mat.ethz.ch (S.H.)

S1 Characteristic force at finite temperature

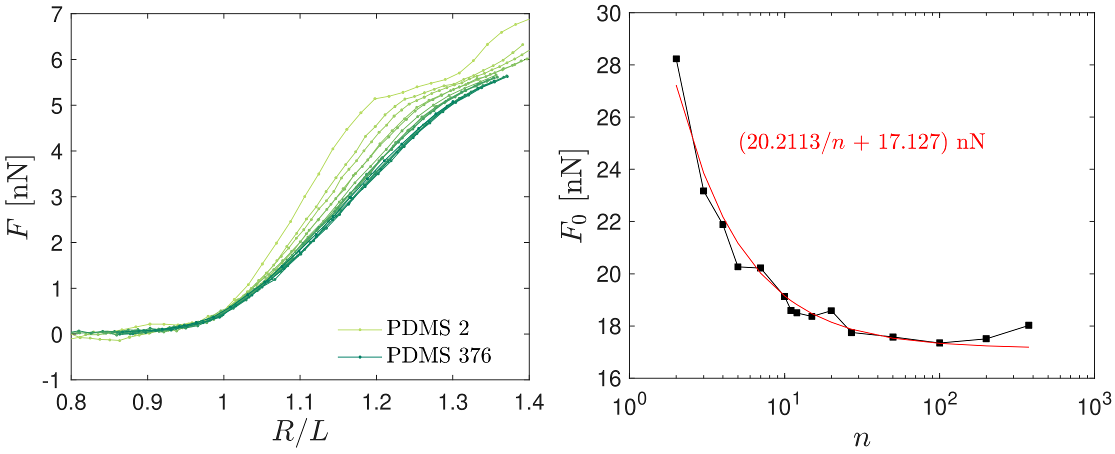

Figure S1: Characteristic force (equivalent to Fig. 1(d) in the main manuscript) at $T = 300\,\mathrm{K}$. The end effect (decreasing F_0 with increasing n) is also observed at finite temperature. Added noise due to entropic contributions impede the characterization of $F_0(n)$ within the enthalpic region, for which we still recover $F_0 \propto (n+1)/n$. In combination with a study of bond length distributions along the chain (for all forces in the enthalpic part, the two terminal Kuhn springs (Si-Si-Si) are longer than all internal springs), this leads us to the non-uniform chain of springs model.

S2 Kuhn length

We obtained an independent estimate of the Kuhn length of the PDMS chains by analyzing the Si-Si vector correlation function $C(i) = \langle \mathbf{u}_j \cdot \mathbf{u}_{j+i} \rangle$, where $\mathbf{u}_j$ denotes the unit vector parallel to the vector connecting the jth and $(j+1)$th Si atom along the PDMS backbone, and the average is taken over all $j \in \{1, .., n\}$ within an ensemble of equilibrium PDMS-n chains. For the FRC model, $\ln C(i) = -\ell/L_p$, where L_p is the persistence length, and $\ell \approx 2.932 \pm 0.003\,\text{Å}$ the measured average distance between adjacent Si atoms. We obtain $L_p = 2.67 \pm 0.07\,\text{Å}$ ($n = 10$), $L_p = 2.86 \pm 0.09\,\text{Å}$ ($n = 20$), $L_p = 2.69 \pm 0.06\,\text{Å}$ ($n = 50$), $L_p = 2.77 \pm 0.05\,\text{Å}$ ($n = 100$). The Kuhn length L_k is twice as large as the persistence length, confirming $L_k \approx 5.5\,\text{Å}$. This is in agreement with prior literature estimates.[61]

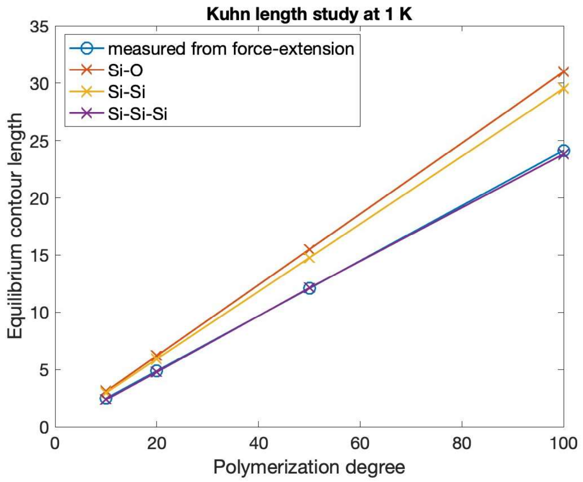

Figure S2: Measured equilibrium contour length (blue circles) in comparison to different models of Kuhn segments (Si-O, Si-Si, and Si-Si-Si). Using force-extension simulations at $T = 1\,\mathrm{K}$, the measured equilibrium contour length (specified in units of nm) follows from the intercept of the enthalpic part on the horizontal axis at $F = 0$. Following the EFJC model, the equilibrium contour length is the sum of all Kuhn segment lengths. We find that taking Si-O or Si-Si as a Kuhn segment overestimates the measured equilibrium contour length, while taking Si-Si-Si as the elementary Kuhn unit is in good agreement with measured values.

S3 Parameters of the double well potential

Table SI: Parameters of the effective bond potential $U(b; F)$ given by Eq. (4). All results have been produced using the stated values, whose error is less than 3% for k_2 and c_2, and less than 1% for b_1 and b_2. For PDMS 4 (4 Si-O bonds) we obtained $\tau_4(F = 90$ kcal.mol^{-1}.Å^{-1} $= 6.2531$ nN$) = 0.221 \pm 0.002$ ns from atomistic simulation at $T = 300$ K. This value is reproduced via Brownian dynamics using $\zeta = 105$ ng/m^2. For linked PDMS 4 (2 Si-C bonds) $b_r = 0.255$ nm and we obtained $\tau_2(F = 90$ kcal.mol^{-1}.Å$^{-1}) = 0.70 \pm 0.04$ ps and $\tau_1(F = 80$ kcal.mol^{-1}.Å$^{-1}) = 0.94 \pm 0.05$ ps and $\tau_1(F = 75$ kcal.mol^{-1}.Å$^{-1}) = 51.3 \pm 0.8$ ps from atomistic simulation at $T = 300$ K. This value is reproduced via Brownian dynamics using $\zeta = 0.002$ ng/m^2. $^{(*)}$ literature estimate.

System	b_r [nm]	k_2 [kg.nm^{-2}.s^{-2}]	c_2 [kg.s^{-2}]	b_2 [nm]	b_1 [nm] $\tilde{F} \equiv F/$ nN
PDMS 4 (8 Si-O bonds)	0.22	579866	-55.9156	0.186	$0.15471 + 0.00085126\,\tilde{F} + 0.0002648\,\tilde{F}^2$
PDMS 376 (752 Si-O bonds)	0.22	621285	-41.419	0.185	$0.15471 + 0.00085126\,\tilde{F} + 0.0002648\,\tilde{F}^2$
linked PDMS 4 (2 Si-C bonds)	0.255	0	186	0	$0.20042 + 0.0029917\,\tilde{F} + 0.00031558\,\tilde{F}^2$
linked PDMS 4 (1 C-C bond)	$^{(*)}$0.181	0	800	0	$0.151 - 0.0005\,\tilde{F}$

S4 Mean bond rupture time

Consider a Brownian bond whose time-dependent length $b(t)$ resides within the interval $[b_0, b_r]$. The bond is assumed to change its length resulting from three types of forces: Deterministic forces due to a one-dimensional potential $U(b; F)$ of mean force, which we determine from atomistic simulation at constant force F, a frictional force (friction coefficient ζ) resulting from the surrounding medium, and a stochastic force whose strength is governed by the fluctuation-dissipation theorem. The Langevin equation for the bond length b thus reads[53]

$$\frac{d}{dt}b = -\frac{1}{\zeta}\frac{dU(b;F)}{db} + \sqrt{\frac{2k_\mathrm{B}T}{\zeta}}\eta(t), \tag{S-1}$$

where $\eta(t)$ represents uncorrelated white noise, $\langle\eta(t)\rangle = 0$ and $\langle\eta(t)\eta(t')\rangle = \delta(t-t')$. We further consider an adsorbing boundary at $b = b_r$ (the rupture bond length) and a reflecting boundary at $b = b_0$. Let the conditional probability $p_2(b', t|b, 0)$ distribution capture the probability that a bond, whose length is b at time 0, assumes length b' at a later time $t' \geq 0$, with $b, b' \in [b_0, b_r]$. Inline with our assumptions, $p_2(b', t|b, 0)$ solves an adjungated Fokker-Planck equation corresponding to the Langevin Eq. (S-1)[54,55]

$$-\frac{\partial}{\partial t}p_2(b',t|b,0) \;=\; -\left[-\frac{1}{\zeta}\frac{\partial U(b;F)}{\partial b}\frac{\partial}{\partial b} + \frac{k_\mathrm{B}T}{\zeta}\frac{\partial^2}{\partial b^2}\right]p_2(b',t|b,0) \tag{S-2}$$

subject to initial condition $p_2(b', 0|b, 0) = \delta(b' - b)$ and the abovementioned constraints. Then

$$G(b_r, t|b) = \int_{b_0}^{b_r} p_2(b',t|b,0)\, db' \tag{S-3}$$

is the probability that $b(t)$ resides within the interval $[b_0, b_r]$ at time t. The G is thus not normalized except in the limit $b_r \to \infty$, i.e., one has $G(\infty, t|b) = 1$. Further $G(b_r, 0|b) = 1$ since $p_2(b', 0|b, 0) = \delta(b' - b)$, and $G(b_r, \infty|b) = 0$, since the bond length exceeds b_r with a nonzero probability. One can write down an equation for G based on the equation (S-2) for p_2.[53] Due to the boundary conditions for p_2, the boundary conditions for G read $G(b_r, 0|b) = 1$ for $b \in [b_0, b_r]$ and

$G(b_r, 0|b) = 0$ otherwise, and

$$G(b_r, t|b)|_{b=b_r} = 0, \qquad \frac{\partial}{\partial b} G(b_r, t|b)\bigg|_{b=b_0} = 0. \tag{S-4}$$

Because we are interested in the mean rupture time, we introduce the fraction $f(b_r, t|b)$ of bonds that reach b_r (and thus leave the interval $[b_0, b_r]$) within the time interval $[t, t+dt]$. One has

$$-dG(b_r, t|b) = -\partial_t G(b_r, t|b) dt \equiv f(b_r, t|b) dt, \qquad f(b_r, t|b) = -\partial_t G(b_r, t|b). \tag{S-5}$$

The quantity

$$T_1(b_r, b) = \int_0^\infty t f(b_r, t|b) dt = -\int_0^\infty t \partial_t G(b_r, t|b) dt = \int_0^\infty G(b_r, t|b) dt \tag{S-6}$$

is the mean bond rupture time. Higher moments can also be calculated with the cumulative distribution function $G(b_r, t|b)$ at hand. The equation for G can now be used to write down coupled equations for the moments

$$T_j(b_r, b) = \int_0^\infty t^n f(b_r, t|b) = j \int_0^\infty t^{j-1} G(b_r, t|b) \qquad (j \geq 1, T_0 = 1). \tag{S-7}$$

The equation for the jth moment reads

$$\left[\frac{k_B T}{\zeta} \frac{\partial^2}{\partial b^2} - \frac{1}{\zeta} \frac{\partial U(b;F)}{\partial b} \frac{\partial}{\partial b} \right] T_j(b_r, b) = -j T_{j-1}(b_r, b) \qquad j = 1, 2, .. \tag{S-8}$$

and the boundary conditions for $T_j(b_r, b)$ are

$$T_j(b_r, b_r) = 0, \qquad \frac{\partial}{\partial b} T_j(b_r, b)\bigg|_{b=b_0} = 0. \tag{S-9}$$

For $j = 1$ the above Eq. (S-8) reduces to

$$\left[\frac{k_{\mathrm{B}}T}{\zeta}\frac{\partial^2}{\partial b^2} - \frac{1}{\zeta}\frac{\partial U(b;F)}{\partial b}\frac{\partial}{\partial b}\right] T_1(b_r, b) = -1. \tag{S-10}$$

This ordinary differential boundary problem is solved by

$$T_1(b_r, b) = \int_b^{b_r} dz\, \frac{1}{\Psi(z)} \int_{b_0}^{z} \frac{\Psi(y)}{D}\, dy, \tag{S-11}$$

with

$$\Psi(z) = \exp\left[\int^z \frac{-\frac{1}{\zeta}\frac{\partial U(b;F)}{\partial b}}{k_{\mathrm{B}}T/\zeta} db\right] = \exp\left[-\frac{U(z;F)}{k_{\mathrm{B}}T}\right]. \tag{S-12}$$

If we average the mean rupture time over all possible initial lengths of the bond,

$$\overline{T_1}(b_r) = \frac{\int_{b_0}^{b_r} T_1(b_r, b) p_0(b)\, db}{\int_{b_0}^{b_r} p_0(b)\, db}, \tag{S-13}$$

where $p_0(b)$ is the density distribution of the initial value. Within the manuscript we denote the mean rupture time by $\tau(F)$ with

$$\tau(F) = T_1(b_r, b = b_1), \tag{S-14}$$

to highlight its dependency on F, because b_r is a bond type-specific constant, because we are not considering higher moments than the first moment, and because we choose the bond to reside at $t = 0$ in its energetic minimum, located at $b = b_1(F)$. Recall that our potential has the features $U(b_1) = U'(b_1) = 0$ and $U''(b_1) > 0$. Moreover, we use $b_0 = 0$ as the reflecting boundary, noting that the precise choice does not matter as U tends to diverge at $b \to 0$ due to excluded volume interactions. We checked that the $\tau(F)$ calculated semi-analytically via Eq. (S-11) with Eq. (S-12) (numerical integration of the double-integral) is exactly identical with the mean rupture time obtained via Brownian dynamics of the Langevin equation (S-1) with reflecting boundary at $b_0 = 0$, adsorbing boundary at b_r, and initial condition $b(0) = b_1(F)$, if results are extrapolated to infinitely small time step.

S5 Influence of boundary conditions

Explanation of **fix-move**, **fix-force**, **fix-smd** and **fix-spring**. fix-smd is the primary method for chain stretching used within the manuscript. Only Figure 1(c) is obtained using fix-move and fix-force, while all other figures are obtained with fix-smd.

fix-move: move the positions of 2 terminal Si atoms of a PDMS chain with constant velocity V (i.e. stretch a chain with constant velocity V). When V is set to 0, we can fix the extension R of a PDMS chain and measure the force F to obtain force-extension relation.
fix-force: apply a constant force F on a terminal Si atom while fixing the position of the other terminal Si atom (i.e. stretch a polymer chain with constant force F). After equilibrium is reached, we register R to measure force extension.
fix-smd: apply a constant repulsive force F between 2 terminal Si atoms. After equilibrium is reached, we register R to measure force extension.
fix-spring: use 2 springs to stretch the 2 terminal Si atoms of a PDMS chain. We can measure the R and F (forces in 2 springs) to obtain force-extension. However due to the oscillation of springs, the measurement error is extremely large so no results presented in this manuscript are measured with this method.

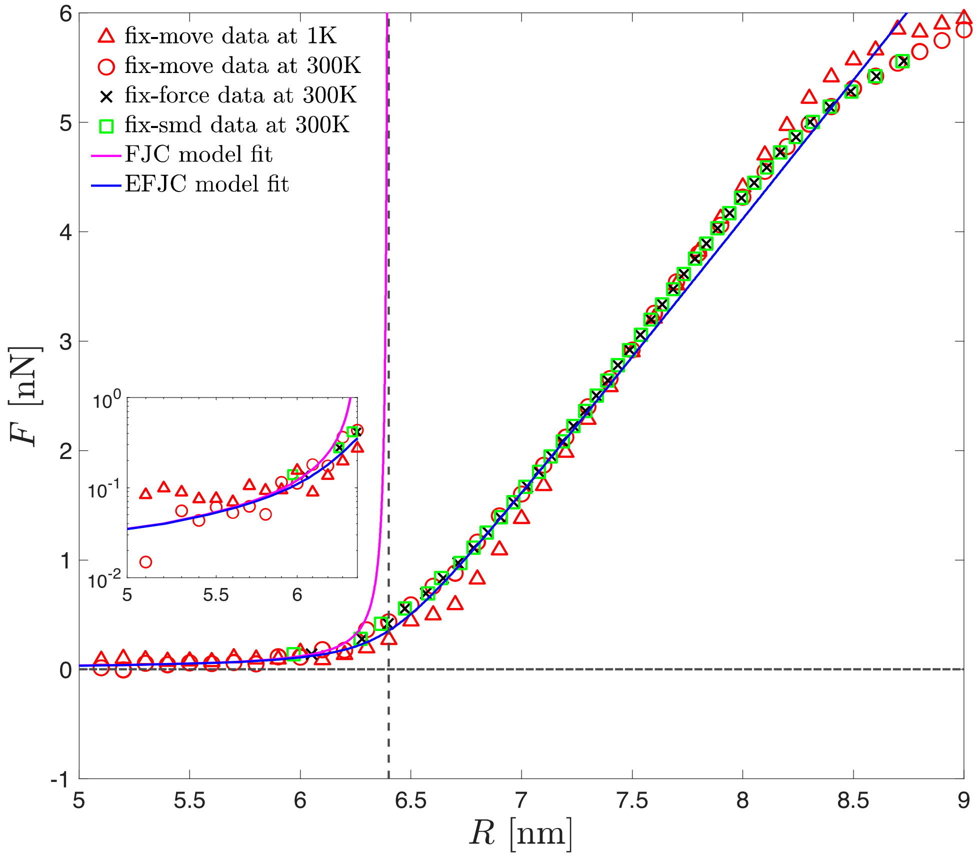

Figure S3: Investigation of the influence of boundary conditions, temperature, and the presence/absence of solvent molecules on the force-extension relation of PDMS-27. We find that the choice of boundary condition does not influence the obtained force-extension relation (fix-spring is omitted, as it is not suitable in force-extension measurements due to large measurement errors linked to spring oscillations). fix-move measurements at $T = 1$K (red triangles) show a slightly smoothed-out transition between entropic- and enthalpic regimes, which stems from reduced entropic contributions.

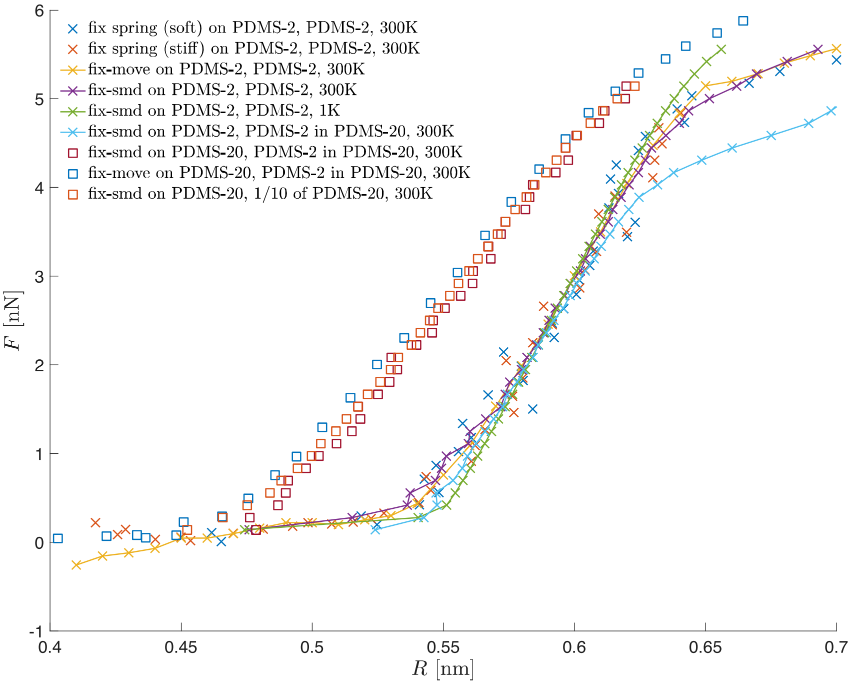

Figure S4: Investigation of the influence of boundary conditions on the observed 'end effect'. We focus on three different molecules: PDMS-2, PDMS-20, as well as a PDMS-2 segment (Si-Si-Si) located at the center of PDMS-20. Different boundary conditions do not influence the force-extension relation within the enthalpic part. Furthermore, stretching an isolated PDMS-2 molecule is equivalent of stretching PDMS-2 at the center of PDMS-20. The difference between data plottes as $\times$ and $\square$ is the chain length between the 2 stretching points.

S6 Bond length distributions and potentials

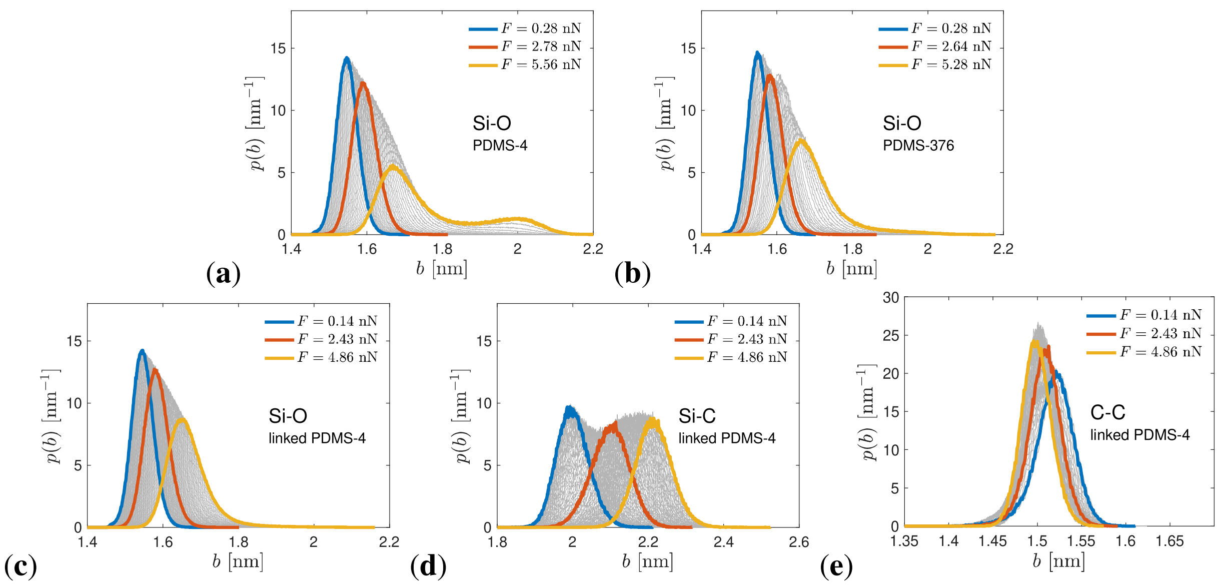

Figure S5: Bond length distribution on (**a**) PDMS-4, (**b**) PDMS-376 and (**c-e**) linked PDMS-4 obtained from atomistic MD. Simulations are performed at $T = 300$ K.

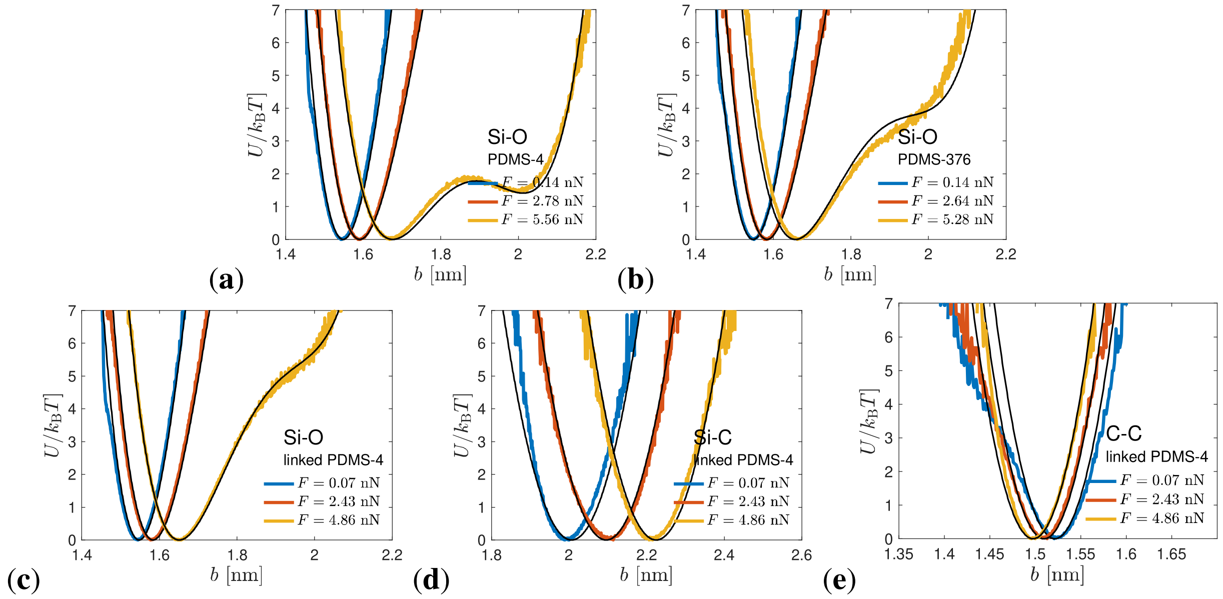

Figure S6: Effective bond potentials calculated from the bond length distributions on (**a**) PDMS-4, (**b**) PDMS-376 and (**c-e**) linked PDMS-4, shown in Fig. S1, along with the Si-O and Si-C fit functions (solid black lines) stated in the manuscript.

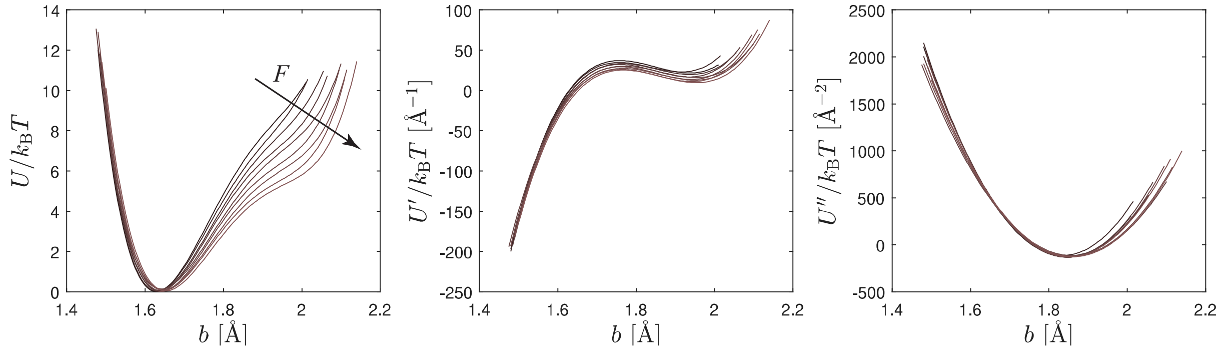

Figure S7: Potential of mean force and its derivatives, $U(b)$, $U'(b)$ and $U''(b)$ for Si-O at different levels of relatively strong applied force $F > 3$ nN (MD simulations performed in the presence of solvent molecules). Since U'' exhibits parabolic and F-independent shape and location, the corresponding parameters b_2, c_2, k_2 in the 4th order polynomial are treated as F-independent constants.

S7 Rupture times

Here, we provide evidence that the measured rupture times are basically unaffected by the presence of HMDSO solvent molecules. In the absence of solvent, the friction coefficient ζ is implicitly captured by the employed thermostat. This finding allows us to simulate the exponential tail of the rupture time distribution in the absence of solvent (Fig. 3a). This renders computatiosn feasible (as it is two orders of magnitude cheaper than the full atomistic simulation of the solvated PDMS chain). Shown in Fig. S8 is the cumulative fraction of ruptured PDMS-6 chains versus time both in the presence and absence of solvent molecules.

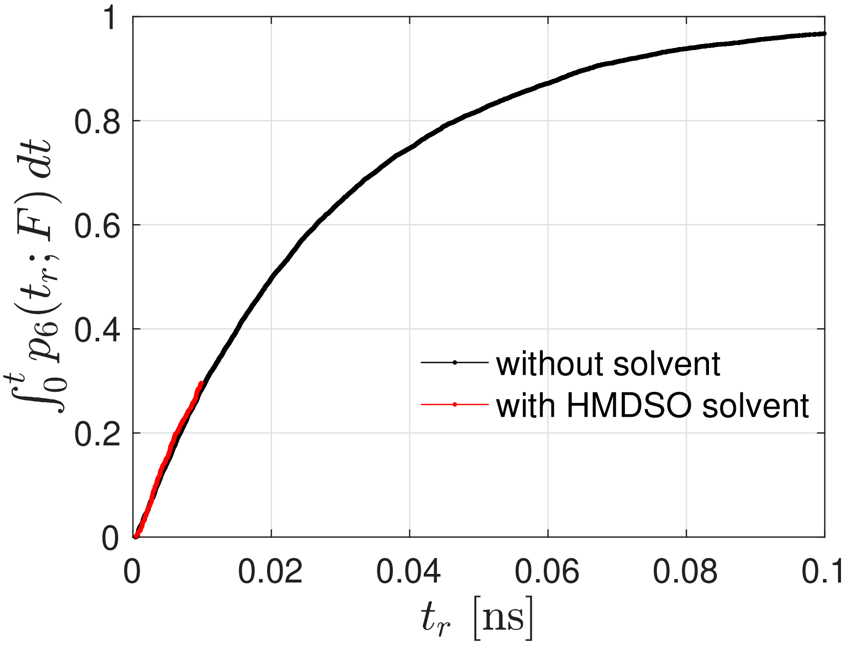

Figure S8: Cumulative rupture time distribution for PDMS-6 at $F = 6.39$ nN. Red: Simulation in the presence of HMDSO solvent molecules. Black: Simulations without solvent. Results obtained by averaging over 100 (with) and 10000 (without solvent) independent start configurations. Simulations are performed at $T = 300$ K.

S8 Mean chain rupture times for different polymerization degrees

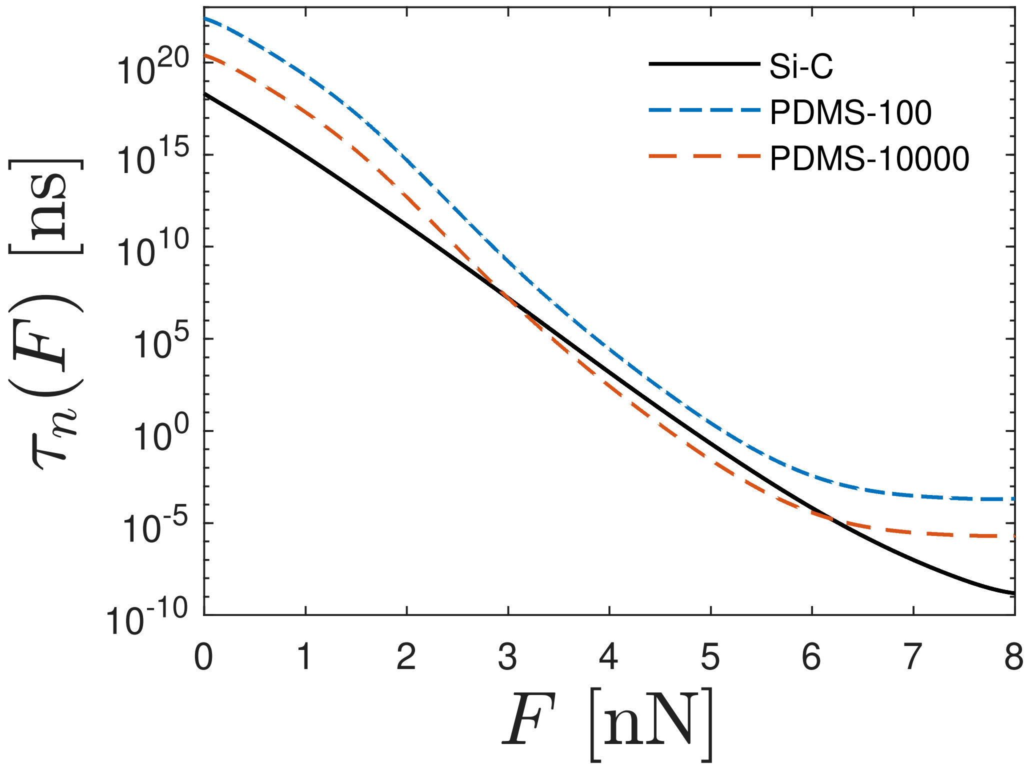

Figure S9: Equivalent of Figure 3c for different polymerization degrees n. At low n (blue dashed line), crosslinking junctions are weaker than Si-O bonds along the backbone (corresponding to the dashed line at position (A) in Figure 4c. A double crossover between Si-C and Si-O bonds is observed at high n (dashed red line). This corresponds the re-entrant effect observed in Figure 4c.
